# Supplementary material for: Content-rich biological network constructed by mining PubMed abstracts
Source: BMC Bioinformatics. 2004 Oct 8;5:147. doi: 10.1186/1471-2105-5-147 (PMC528731; doi:10.1186/1471-2105-5-147)
Supplement: Additional File 2 — The original results of the above study (non-essential files are deleted to keep the file size under the limit set by BMC bioinformatics). [file 1471-2105-5-147-S2.bz2 › chilibotAdditionalFile2/dip20/482ID8702798E10572/html/IL8RA_CXCL5.html]

 


 **IL8RA** and **CXCL5** 
  
Found 14 abstracts in PubMed, retrieved 14.  
 

 What does Google say? 
 PDF only 
| .edu only 

---

**Interactive relationship** (e.g. stimulation, inhibition, etc)

**Neutral relationship**- IL 8 activates neutrophils by binding to two distinct seven transmembrane 7 TMR G protein coupled receptors CXCR1  [ **IL8RA** ]  IL 8RA and CXCR2 IL 8RB, while GROalpha, GRObeta, GROgamma, and ENA 78  [ **CXCL5** ]  bind to and activate only CXCR2.  Ref: 9553055 J Biol Chem, 1998
- From these data, it can be concluded that, in addition to IL 8, GCP 2, but not ENA 78  [ **CXCL5** ] , efficiently binds to both CXCR1  [ **IL8RA** ]  and CXCR2.  Ref: 9692902 Eur J Biochem, 1998
- In contrast, only IL 8 and GCP 2 but not ENA 78  [ **CXCL5** ] , competed for 125I IL 8 binding to CXCR1  [ **IL8RA** ] .  Ref: 9692902 Eur J Biochem, 1998
- GROalpha, GRObeta, GROgamma, NAP 2, and ENA 78  [ **CXCL5** ]  competed weakly for the high affinity IL 8 binding site at  **IL8RA** .  Ref: 8702798 J Biol Chem, 1996
- The rank order of agonist potency, based on inspection of the mean effective concentration values EC50, for IL8RB was GROgamma 1 nM > IL 8 4 nM approximately GROalpha 5 nM approximately GRObeta 4 nM approximately NAP 2 7 nM > ENA 78  [ **CXCL5** ]  11 nM, and for  **IL8RA**  was IL 8 4 nM >>> ENA 78  [ **CXCL5** ]  40 nM approximately NAP 2 45 nM > GROalpha 63 nM approximately GROgamma 65 nM >> GRObeta.  Ref: 8702798 J Biol Chem, 1996
- GCP 2 desensitized the calcium increase induced by IL 8 in both CXCR1  [ **IL8RA** ]  transfected and CXCR2 transfected cells, but ENA 78  [ **CXCL5** ]  only affected the IL 8 induced calcium response in CXCR2 transfectants.  Ref: 9692902 Eur J Biochem, 1998

**Non-interactive relationship** (e.g. studied together, co-existance, homology, etc.)

- Thus in COPD, in addition to CXCL8 and CXCR1  [ **IL8RA** ] ,  **CXCL5**  and CXCR2 appear to play important roles in the airway neutrophilia characteristic of severe exacerbations.  Ref: 12857718 Am J Respir Crit Care MedAm J Respir Crit Care Med, 1998
- ... GCP 2 was tenfold more potent than ENA 78  [ **CXCL5** ]  on CXCR1  [ **IL8RA** ]  transfectants.  Ref: 9692902 Eur J Biochem, 1998
- ... for ENA 78  [ **CXCL5** ] , threefold higher concentrations were necessary to obtain a calcium response in CXCR1  [ **IL8RA** ]  transfected cells than in CXCR2 transfectants.  Ref: 9692902 Eur J Biochem, 1998
- By measuring calcium flux in human embryonic kidney 293 cells transfected with plasmids encoding  **IL8RA**  or IL8RB, we have now defined receptor selectivity for GRObeta, GROgamma, and ENA 78  [ **CXCL5** ] .  Ref: 8702798 J Biol Chem, 1996
- At 10 nM and higher concentrations, GCP 2 and IL 8 induced significant activation of CXCR1  [ **IL8RA** ]  expressing cells, but no activity was found with GRO alpha and ENA 78  [ **CXCL5** ] .  Ref: 9485196 Eur J Immunol, 1998
- RESULTS Flow cytometry and radioligand binding data indicate that IL 8, GCP 2, and ENA 78  [ **CXCL5** ]  equivalently reduced CXCR 1  [ **IL8RA** ]  and CXCR  [ **IL8RA** ]  2 cell surface expression by 34% to 54%.  Ref: 10593335 Arch Surg, 1999
- Using Jurkat cells that stably express either CXCR1  [ **IL8RA** ]  or CXCR2, we studied receptor activation by IL 8, GCP 2 epithelial neutrophil activating protein 2 ENA 78  [ **CXCL5** ]  which shares 77% identical amino acids with GCP 2 and growth regulated oncogene alpha GRO alpha.  Ref: 9485196 Eur J Immunol, 1998
- By comparison with the NSC and S COPD groups, neutrophilia and gene expression for epithelial derived neutrophil attractant 78  **CXCL5** , interleukin 8 CXCL8, CXCR1  [ **IL8RA** ]  and CXCR2 were each up regulated in the E COPD group p&lt.  Ref: 12857718 Am J Respir Crit Care MedAm J Respir Crit Care Med, 1998
- anti ENA 78  [ **CXCL5** ]  antibody and anti CXCR  [ **IL8RA** ]  2 antibody inhibited the chemotaxis.  Ref: 11861308 Blood, 2002
